# Rasch Analysis of the Adult Strabismus Quality of Life Questionnaire (AS-20) among Chinese Adult Patients with Strabismus

**DOI:** 10.1371/journal.pone.0142188

**Published:** 2015-11-06

**Authors:** Zonghua Wang, Juan Zhou, Xingli Luo, Yan Xu, Xi She, Ling Chen, Honghua Yin, Xianyuan Wang

**Affiliations:** 1 School of Nursing, Third Military Medical University, Chongqing, China; 2 Department of Ophthalmology, Daping Hospital, Third Military Medical University, Chongqing, China; 3 Department of Ophthalmology, Southwest Eye Hospital, Third Military Medical University, Chongqing, China; 4 Department of Ophthalmology, Xinqiao Hospital, Third Military Medical University, Chongqing, China; 5 Department of Gastroenterology, the 324th Hospital of PLA, Chongqing, China; School of Ophthalmology and Optometry and the Affiliated Eye Hospital, Wenzhou Medical University, CHINA

## Abstract

**Background:**

The impact of strabismus on visual function, self-image, self-esteem, and social interactions decrease health-related quality of life (HRQoL).The purpose of this study was to evaluate and refine the adult strabismus quality of life questionnaire (AS-20) by using Rasch analysis among Chinese adult patients with strabismus.

**Methods:**

We evaluated the fitness of the AS-20 with Rasch model in Chinese population by assessing unidimensionality, infit and outfit, person and item separation index and reliability, response ordering, targeting and differential item functioning (DIF).

**Results:**

The overall AS-20 did not demonstrate unidimensional; however, it was achieved separately in the two Rasch-revised subscales: the psychosocial subscale (11 items) and the function subscale (9 items). The features of good targeting, optimal item infit and outfit, and no notable local dependence were found for each of the subscales. The rating scale was appropriate for the psychosocial subscale but a reduction to four response categories was required for the function subscale. No significant DIF were revealed for any demographic and clinical factors (e.g., age, gender, and strabismus types).

**Conclusion:**

The AS-20 was demonstrated by Rasch analysis to be a rigorous instrument for measuring health-related quality of life in Chinese strabismus patents if some revisions were made regarding the subscale construct and response options.

## Introduction

Adult strabismus, with an estimated prevalence of 4% [1], is often accompanied by ocular misalignment, blurred vision, diplopia, and eyestrain. In addition to the functional complaints, strabismus could also decrease patients’ quality of life (QoL) by adversely affecting psychological and social functioning. Previous studies showed that adult strabismus patients have suffered from anxiety and depression, low self-esteem, lack of confidence, limited job opportunities and difficulties with interpersonal relationships [2–4]. Consequently, they are likely to avoid social contacts, abandon outdoor activities, and develop mannerisms to hide the defects.

In recent years health-related QoL (HRQoL) among Chinese adult patients with strabismus have received increasing concerns from researchers and clinical professionals. Except for one study exploring improvements on psychosocial functioning after strabismus surgery [5], other studies have focused on translation and culturally adaptation of vision-related QoL (VRQoL) instruments [6–10] that may be applicable to strabismus patients. Our previous studies have translated the original adult strabismus-20 (AS-20) into Chinese. We chose the AS-20 since it had the advantages over other VRQoL scales for being developed specifically to strabismus and showed satisfactory reliability (Cronbach’s α = 0.94) and validity [11]. The Chinese version of the AS-20 was demonstrated by traditional psychometric methods (classical test theory, CTT) that it was a reliable (Cronbach’s α = 0.91) and valid tool in Chinese population [12] after some item deletions and re-categorization.

Rasch model has been recently used to investigate psychometric properties of the AS-20 in American and India populations [13,14]. Both studies found the lack of unidimensionality in the full-length of the AS-20, and indicated the needs for reduction of response categories and for deletion of several misfitting items. However, no Rasch analysis of the AS-20 in Chinese settings has been reported. One benefit of Rasch analysis is that it considers both the difficulty of each item and the latent trait of subjects to provide interval-level estimates which increase statistical power and minimize the cost of clinical research by significantly reducing the sample size required. In contrast, the traditional methods (CTT) has the limitation of assuming each item contributes equally to the overall assessment of the latent trait. These assumptions of uniform weighting between response options for each item may not be true [14,15]. Moreover, Rasch model provides a deeper insight into the underlying features, such as unidimensionality, items fit and targeting, and response ordering. These are essential for an instrument to make meaningful comparisons of latent trait between different groups, or to compare across time [15]. Given these advantages of Rasch model, this study aims to investigate the psychometric properties of the AS-20 by using Rasch analysis among adult strabismus patients in the context of Chinese culture.

## Methods

Study cohort were 247 Chinese adult patients with strabismus attending three tertiary hospitals in Chongqing during April and September 2014. The strabismus patients who met the following criteria were invited to take part in the study [12]: 1) aged 18 years and over; 2) no history of any eye-related surgery or any diagnosed emotional disorders; 3) no other facial or ocular comorbidities or any acute eye diseases; and 4) the angle of deviation by prism at distance was no less than 15pd. All data were collected prior to any strabismus-related surgery. Information about demography and clinical features were also collected.

Ethical approval was obtained from the human ethics committee of Third Military Medical University. The whole study was in accordance with the Declaration of Helsinki. No written consent were collected, and the consent to participate in this survey was assumed upon the completion of this questionnaire (i.e., completing the questionnaires implies giving consent to participate). The participants were left alone in a reception room to complete the Chinese version of the AS-20 after patient information sheet (PIS) and full introduction have been given.

### Statistical analysis

Descriptive analysis of demography and clinical features were performed by SPSS software (IBM, version 21.0). Winsteps^®^ software (version 3.81.0) was adopted to conduct Rasch analysis.

### Rasch Analysis

In this study, we began with a test of dimensionality by performing the principal component analysis (PCA) of the residuals. The residuals are those parts of the data not explained by the Rasch model. Unidimensionality could be considered if the PCA results show raw unexplained variance by the first contrast with an eigenvalues smaller than 2.0 [16].

An assessment of local item independence and item fit mean square was conducted to further examine how each item fit the scale to measure the underlying trait. Intra-item standardized residual correlations of > 0.7 were used to identify high local dependence between items, which indicates that these items may duplicate some feature of each other or they both incorporate some shared dimension. Such items should be considered of combining or eliminating. If a MnSq value of an item places between 0.7 and 1.3 [16], it indicates that the item contributes to a single underlying construct (unidimensionality). Otherwise, the patients may respond to the item erratically, i.e., it isn’t always ‘harder’ (or ‘easier’) for all patients. Generally such misfitting item should be removed. The standardized Z-score (ZSTD) is a t-statistic to report the statistical significance (probability) of the MnSq value; in this study, the ZSTD were presented but not considered for analyzing item fitness because these statistics were sample-dependent and may elevate as sample size increases [15].

The differential item functioning (DIF) provides an indicator to investigate whether items show different difficulty estimates across subgroups [17]. The demographic and clinical variables we selected for DIF analysis included: gender, age (≤ median age [29.69 years] vs. > median age), education level (below/ above high school), living areas (urban/ rural), social support (always/ sometimes & none), strabismus type (exotrapia/ esotropia), diplopia (presence/ absence) and health insurance (presence/ absence). A DIF contrast < 0.50 logits was defined as small or absent, DIF 0.50 to 1.0 as minimal (inconsequential), and DIF > 1.0 as notable [17,18].

Then we inspected person and item separation and reliability (PSEP) and person-item maps to evaluate precision and targeting within each subscale. The minimum acceptable value of person separation is 2.0 in order to attain the desired level of reliability of at least 0.80. Item separation is used to confirm the item difficulty hierarchy (= construct validity). A desired value of item separation is ≥ 3 (reliability value ≥ 0.9), which implies that the sample size is large enough to confirm the item difficulty hierarchy of the instrument. The targeting refers to whether the latent trait (in this study, i.e., patients’ strabismus-related QoL) matched with the item discrimination (difficulty). For a well-targeted instrument, the difference between the person and item means on the person-item map should be less than 1.0 logits [19].

The category probability curves (CPC) were also examined to explore appropriateness of the response options [20]. The response options in the original AS-20 were a five-point Likert scale from 0 (always) to 100 (never). The CPC showed the range of QoL for which each of the five response categories were most likely to be chosen. Disordered categories indicate that neighboring categories may be indistinguishable to respondents and could be merged [21].

## Results

### Demographic and Clinical Characteristics

A cohort of 247 adult patients with strabismus (mean age, 29.69 ± 11.07 years) completed the AS-20. One hundred twenty-two (49.4%) were female and 107 (43.3%) were living in rural areas. Less than a quarter of the patients (n = 54) hold a university certificate or above, while 104 graduated from high school, 69 from middle school and 20 from primary school. More than one-third of the patients (n = 95) reported that they had often received support from family members and friends. Almost two-thirds (n = 168) had no health insurance to cover strabismus-related treatment and they had to pay for themselves. Regarding clinical features, over a third (n = 90) complained a symptom of double vision. One hundred and seventy-three (70.0%) were diagnosed as exotropia, 74 (30.0%) were esotropia. All patients were in hospital for a strabismus-related corrective surgery for the first time.

### Unidimensionality

**Fig 1:**
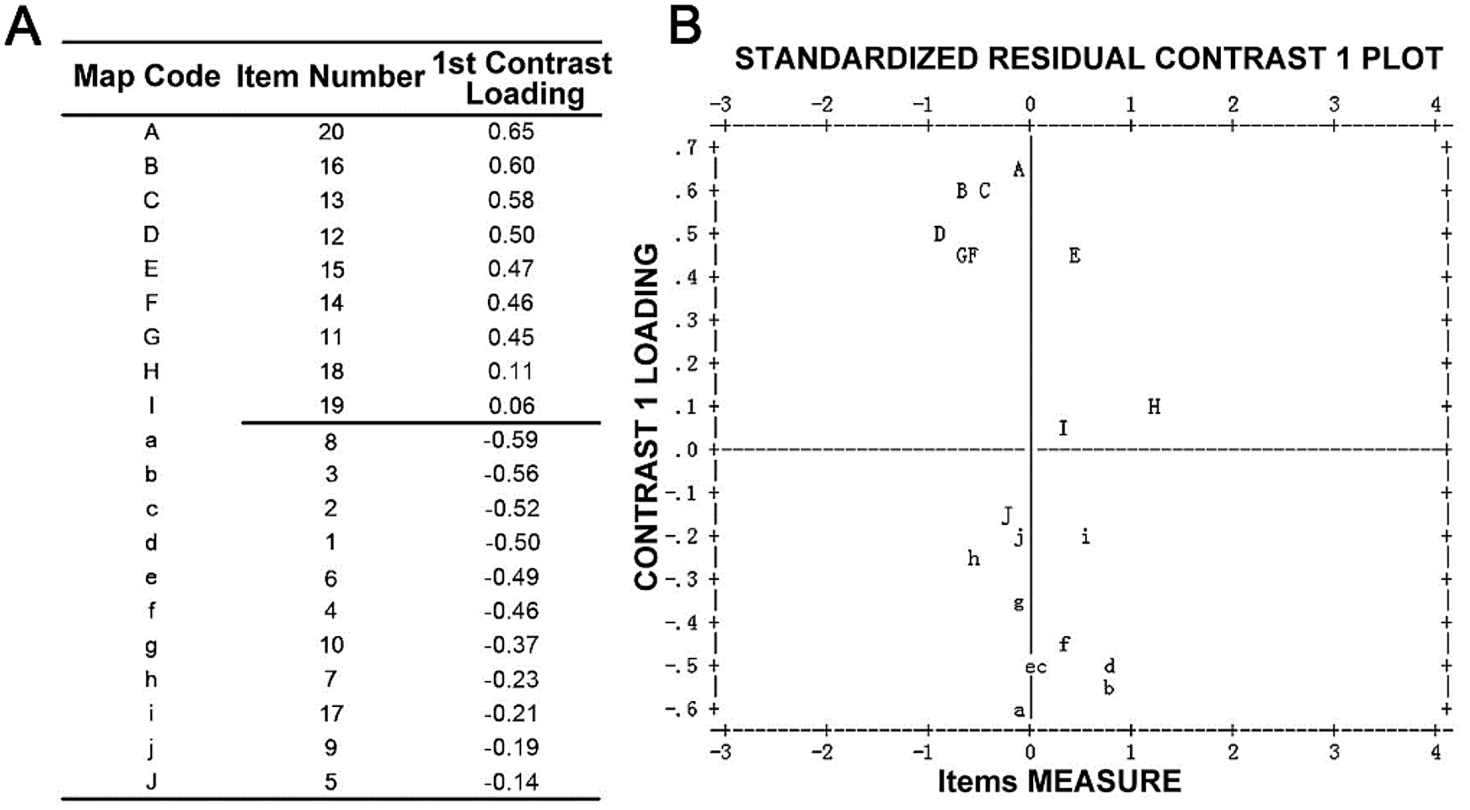
Plot of PCA of residuals analysis.

The PCA results of the overall AS-20 revealed that 46.3% of the total raw variance was explained by the measures and the overall raw unexplained variance explained by the first contrast had an eigenvalue of 3.9 (> 2.0) (Table 1), suggesting a second dimension. Mostly consistent with the original AS-20, the two subscales—the ‘psychosocial subscale’ and the ‘function subscale’- with the same containing items were presented (Fig 1), except for the item 17 ‘I feel stressed because of my eyes’ having been re-categorized into the psychosocial subscale instead of the function one. A second PCA was conducted separately in the newly identified subscales, both suggesting a feature of unidimensionality (Table 1): in the psychosocial subscale, 56.9% of the raw variance was explained by the measures, and 7.3% of the unexplained variance was explained by the first contrast, with an eigenvalue of 1.9; in the function subscale, 52.6% of the raw variance was explained by the measures and the unexplained variance by the first contrast was 1.6 eigenvalue units. All the analyses afterwards were based on the Rasch-revised subscales.

**Table 1:** Dimensionality analysis of the overall AS-20 and subscales.

	Overall scale		Psychosocial (Item 1–10 & Item 17)		Function (Item 11–16 & Item 18–20)	
	Eigen	%	Eigen	%	Eigen	%
**Total raw variance**	37.2	100.0	25.7	100.0	19.0	100.0
explained by measures	17.2	46.3	14.7	56.9	10.0	52.6
explained by persons	6.1	16.5	7.3	28.2	3.9	20.4
explained by items	11.1	29.7	7.4	28.7	6.1	32.2
**Raw unexplained variance (total)**	20.0	53.7	11.0	42.7	9.0	47.4
1st contrast	3.9	10.6	1.9	7.3	1.6	8.3
2nd contrast	1.8	4.9	1.4	5.4	1.4	7.4
3rd contrast	1.6	4.2	1.3	5.0	1.2	6.3
4th contrast	1.2	3.3	1.1	4.4	1.1	5.9
5th contrast	1.1	3.0	1.1	4.1	1.0	5.2

### Local Item Dependence and Item Fit

The intra-item correlations of standardized residuals were between -0.15 and -0.33 in the psychosocial subscale and between -0.11 and -0.28 in the function subscale (Table 2). These results indicated that these items were locally independent (Corr. < 0.7). All items fit the Rasch model well since they have showed satisfactory infit and outfit MnSq values within the range of 0.71 and 1.30 (Table 3); therefore no item in both subscales was deleted.

**Table 2:** Local Item Dependence of Psychosocial and Function Subscales.

Psychosocial subscale			Function subscale		
Item No.	Item No.	Correlation of Residuals	Item No.	Item No.	Correlation of Residuals
**1**	10	-0.33	19	20	-0.28
**1**	7	-0.26	13	18	-0.26
**4**	8	-0.23	15	19	-0.22
**3**	5	-0.22	12	18	-0.22
**4**	5	-0.22	14	16	-0.21
**1**	5	-0.21	16	19	-0.20
**3**	9	-0.21	13	15	-0.18
**7**	17	-0.21	12	15	-0.18
**2**	5	-0.20	12	19	-0.17
**2**	6	-0.20	13	19	-0.17
**3**	17	-0.20	11	18	-0.16
**2**	9	-0.20	14	18	-0.16
**3**	6	-0.19	14	15	-0.16
**2**	8	-0.19	16	18	-0.13
**4**	6	-0.18	15	18	-0.13
**4**	7	-0.17	11	19	-0.13
**3**	10	-0.16	13	20	-0.11
**6**	9	-0.16	11	20	-0.11
**1**	9	-0.16	11	15	-0.11
**4**	17	-0.15	18	20	-0.11

**Table 3:** Infit and Outfit MnSq of Psychosocial and Function Subscales.

Item No.	Infit		Outfit	
	MnSq	ZSTD*	MnSq	ZSTD*
**Psychosocial subscale**				
**10**	1.28	3.0	1.30	2.7
**5**	1.17	2.1	1.26	2.3
**9**	1.05	0.6	1.24	2.3
**1**	1.07	1.0	1.12	1.3
**4**	1.02	0.3	1.05	0.6
**7**	0.95	-0.7	1.06	0.6
**3**	0.98	-0.2	0.94	-0.6
**8**	0.93	-0.9	0.83	-1.7
**17**	0.88	-1.4	0.88	-1.3
**6**	0.87	-1.5	0.81	-2.0
**2**	0.74	-3.2	0.71	-3.3
**Function subscale**				
**19**	1.21	2.4	1.23	2.2
**14**	1.19	1.6	1.02	0.2
**13**	1.15	1.4	1.05	0.4
**11**	1.01	0.1	1.14	0.9
**12**	1.14	1.1	1.06	0.4
**16**	1.00	0.0	0.82	-1.2
**15**	0.89	-1.4	0.99	-0.1
**18**	0.91	-1.0	0.97	-0.3
**20**	0.90	-1.0	0.81	-1.7

* the ZSTD were presented but not considered for analyzing item fitness because these statistics were sample-dependent and may elevate as sample size increases.

### Differential Item Functioning

All the DIF contrast were below 1.0 logits for all the selected variables, indicating that no items had notable DIF (S1 Dataset).

### Overall Performance of Psychosocial Subscale

**Fig 2:**
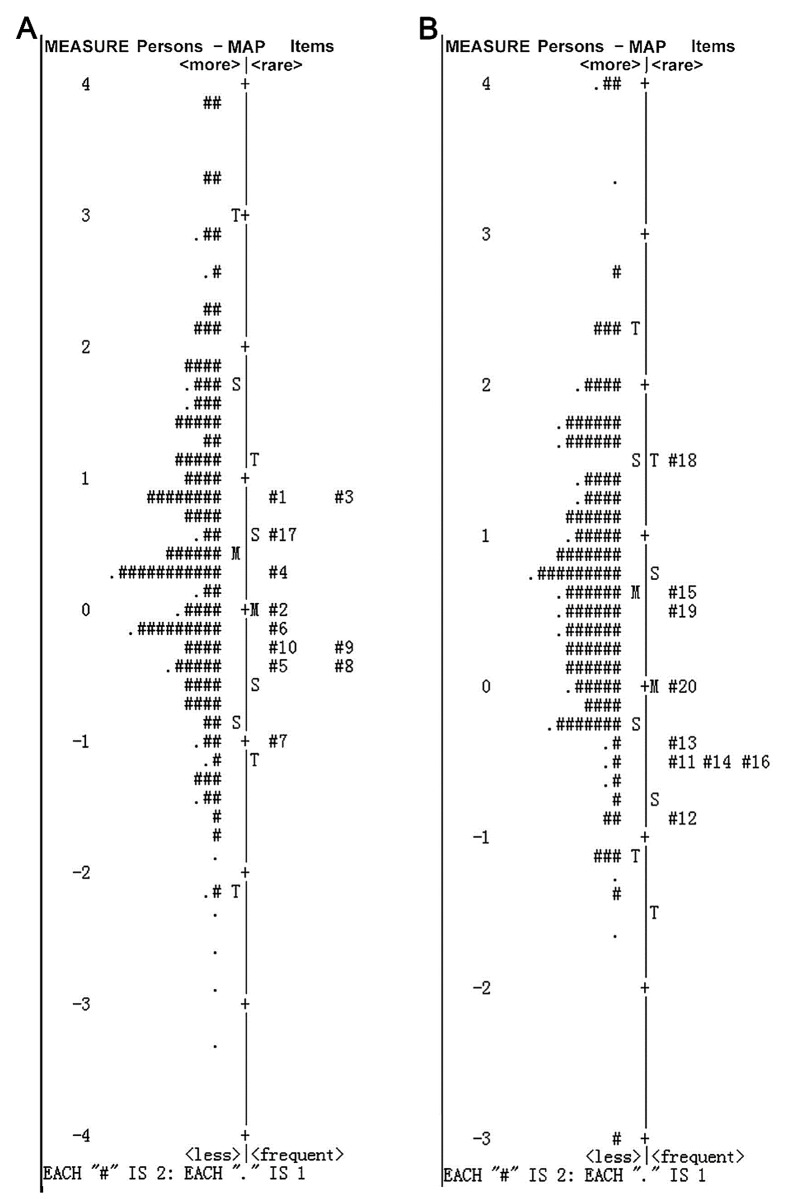
Person-item maps for (A) psychosocial subscale, (B) function subscale. The item discrimination matched well with the strabismus patients’ QoL for both subscales. M, Mean; S, 1 standard deviation; T, 2 standard deviations.

**Fig 3:**
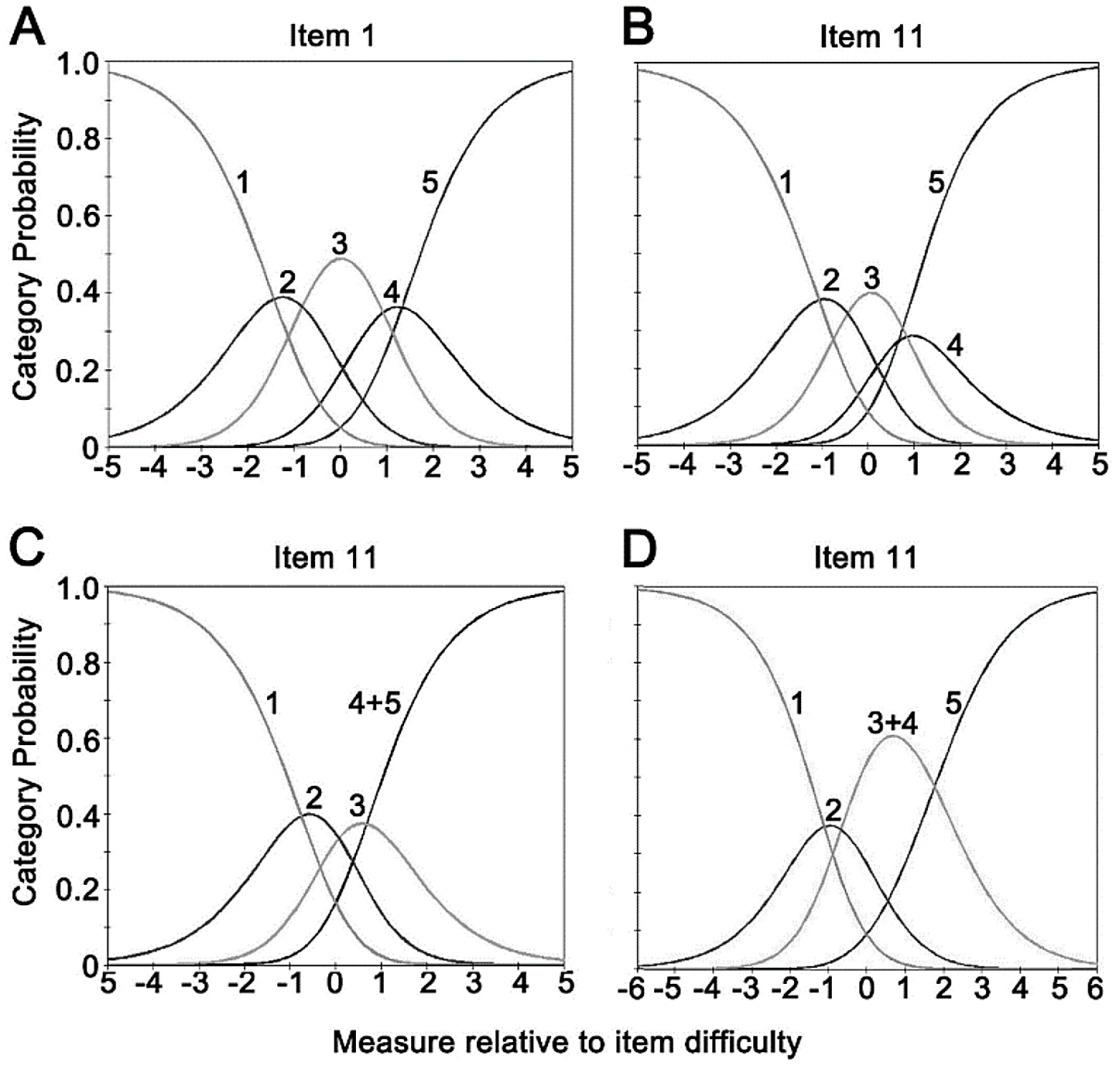
The category probability curves for (A) 5-response psychosocial subscale (representative item #1), (B) 5-response function subscale (representative item #11), (C) 4-response function subscale after the ‘rarely’ and ‘never’ response options were combined, (D) 4-response function subscale after the ‘rarely’ and ‘sometimes’ response options were combined. All curves at the extreme left represents ‘always’ and at the extreme right represents ‘never’. Response categories were properly oriented and distributed for all items in the psychosocial subscale (A). Using 5 response options for the function subscale, it was evident that the ‘rarely’ response was underutilized (B). When the ‘rarely’ option was combined with either the ‘never’ or the ‘sometimes’ option in the function subscale, response categories were properly oriented and distributed (C & D).

The revised psychosocial subscale consisted of the item 1–10 and the item 17. The person separation index (PSI) was 2.70, with a reliability value of 0.88. The item separation index (ISI) was 7.16, with the reliability of 0.98 (Table 4). The difference between the person and item means was less than 1.0 logits (0.45 ± 1.27 logits for mean person vs. 0.00 ± 0.57 logits for mean item; Fig 2A), which suggested good targeting that the item difficulty matched well with the participants’ HRQoL. No disordered response categories were evident (Fig 3A), indicating each category properly represented stepwise increase in severity and the difference between categories on severity could be correctly endorsed by the respondents. Therefore the five-level Likert scaling was retained.

**Table 4:** Overall Performance of Psychosocial and Function Subscales.

	Ideal values	Psychosocial	Function*	Function^#^	Function^§^
**Number of Items**	N/A	11 (items 1–10 and item 17)	9 (items 11–16 and items 18–20)	9 (items 11–16 and items 18–20)	9 (items 11–16 and items 18–20)
**PSI**	≥ 2.0	2.70	1.88	1.53	1.85
**PSI reliability**	≥ 0.80	0.88	0.78	0.70	0.77
**ISI**	≥ 3.0	7.16	9.74	8.31	8.66
**ISI reliability**	≥ 0.90	0.98	0.99	0.99	0.99
**Mean person**	N/A	0.45 ± 1.27	0.67 ± 1.12	1.25 ± 1.26	1.04 ± 1.43
**Mean item**	N/A	0.00 ± 0.57	0.00 ± 0.72	0.00 ± 0.84	0.00 ± 0.90
**the difference between the person and item means**	< 1.0	0.45	0.67	1.25	1.04

* the ‘rarely’ response option was not combined with the ‘never’ response option in the function subscale (the original 5-level response option)

^#^ the ‘rarely’ response option was combined with the ‘never’ response option in the function subscale (the combination of the category 4 and 5)

^**§**^ the ‘rarely’ response option was combined with the ‘sometimes’ response option in the function subscale (the combination of the category 3 and 4)

### Overall Performance of Function Subscale

The revised function subscale consisted of the item 11–16 and the item 18–20. The PSI was 1.88 (< 2.0), with a reliability of 0.78 (< 0.80). The ISI was 9.74, with a reliability coefficient of 0.99 (Table 4). The item difficulty matched well with the participants’ HRQoL (0.67 ± 1.12 logits for mean person vs. 0.00 ± 0.72 logits for mean item; Fig 2B). For response ordering, the CPC illustrated that the category 4 ‘rarely’ response was underutilized (Fig 3B), resulting in disordered thresholds. We combined this category with either the category 5 (‘never’) and the category 3 (‘sometimes’) to find out which one could result in the best measurement precision by PSI and targeting. As shown in Fig 3C and 3D, both the revised four response categories were properly oriented; but the precision and targeting decreased after the combination of response options, with the PSI of 1.53 (reliability = 0.70) and 1.85 (reliability = 0.77) separately and the targeting values over 1.0 logits (Table 4). The combination of the category 4 and the category 3 was chose considering the better PSR than the other combination.

## Discussion

This study was the first Rasch analysis to examine the psychometric properties of the full length of the AS-20 in Chinese adult patients with strabismus. Several findings were highlighted. First, no item was deleted in the overall AS-20 and subscales but a slight change has been made on the subscale form. Second, although the overall AS-20 failed to show unidimesionality, this was obtained in both Rasch-revised psychosocial subscale and function subscale. It is suggested to provide individual score of each subscale instead of obtaining a total score. Third, no misfit item and satisfactory targeting have been found; however, the function subscale did not fit the Rasch model well by showing inadequate measurement precision and underutilized response categories.

This study indicated no item deletion but a form change that the item 17 should be re-categorized into the psychosocial subscale. The re-categorization were also reported in our previous study of the AS-20 by CTT approach [12]: the factor analysis suggested deletion of two items since they revealed equivalent loading in two factors; and three items (including the item 17) originally from the function subscale were re-grouped into the psychosocial subscale considering both factor loading and cultural difference. In another study of the Chinese version of the AS-20, Yu et al. [10] have identified six principal factors without deleting an item, but these factors could be regrouped into two subscales, with all of the 20 items loading in the same subscale as the original AS-20. Each item was correlative with their respective subscale and the Cronbach’s α ranging from 0.819 to 0.883 for the overall AS-20 and the two subscales. Additionally, it was also reported by Rasch analysis that deletion of items and re-construction could improve the AS-20 rigorousness. The earliest Rasch study of the AS-20 was conducted by Leske and colleagues in American strabismus patients [14]. They found both the subscales lacked unidimensionality and recommended formation of four new subscales; but two of these showed low measurement precision and did not function well. A new component of self-perception was divided from the psychosocial subscale, while a second dimension of reading function was divided from the function subscale. Recently Gothwal et al. have conducted Rasch analysis of the Indian version of the AS-20 in 584 Indian adult patients with strabismus [13]. They found that the AS-20 had adequate precision (PSR = 0.87) and targeting but failed to achieve unidimensional unless they deleted six multi-dimensionality causing items and an additional three misfitting items. Overall, these studies indicated that the AS-20 was a reliable and valid questionnaire; however, the inconsistency of the AS-20 item number and subscale form in different cultures and ethnic groups suggested that the AS-20 in its current form was not perfect for its worldwide application. The culturally-appropriate translation and validation of the original AS-20 is required before performing in a new culture and ethnic population.

The reason for the need of deletion and re-categorization of the AS-20 may lie in that the HRQoL is a culture-dependent concept, thus the interpretation of HRQoL varies in different ethnic populations and cultures. In the development of the original AS-20, the function subscale included items relating to physical and emotional functions, while the psychosocial subscale were related to psychosocial functioning and self-awareness [11]. Emotional functions represented a health status without the expression of anxiety and depression; while psychosocial functioning was a broader concept, which stand for not only good psychological health, but also harmonious relationship with nature and social environment [22]. The re-categorization of the item 17 (‘I feel stressed because of my eyes’) may suggest the stress associated with strabismus could go beyond emotional functions and extend to psychological and social functioning in Chinese strabismus patients. Therefore they interpreted the item 17 more relevant to psychosocial functions rather than emotional complaints.

Another reason we assume is that HRQoL is a complicated term covering six aspects of physical, social, and psychological functioning, role activities, overall life satisfaction, and perceptions of health status[23]. Establishing two subdomains ‘psychosocial’ and ‘function’ might not be able to represent all other aspects of HRQoL. Although it indicated excellent reliability and validity in previous studies by using CTT methods, it was because careful clinical and psychometric criteria have been followed during the development of the AS-20. Besides, it might be improper for the AS-20 to include both physical and emotional functions together into the function subscale. For example, the item 17 (I feel stressed because of my eyes) and the item 18 (I worry about my eyes) were related to patients’ emotions while the item 14 (I have problems with depth perception) was related to symptoms. It was proved by the previous studies by using CTT and Rasch model that both methods indicated that the item 17 originally from the function subscale should be re-categoried into the psychosocial subscale. In terms of the psychosocial subscale, it also included items in different aspects (self-perception, social interaction, and psychological status) together. Therefore we assume that some new subdomains should be separated and established from the original two subscales and adding some items to cover more aspects of HRQoL might help improve the AS-20 performance. This assumption was supportive since that Yu et al. extracted six factors in PCA [10] and Leske et al.’s Rasch analysis indicated four new subscales [14].

The person separation index (PSI) and person separation reliability (PSR) refer to the number of statistically distinct levels of person abilities identified by the instrument [24]. The Rasch-revised psychosocial subscale showed the PSI of 2.70 (reliability, 0.88), indicating satisfactory measurement precision to adequately discriminate different levels of quality of life [16]. In contrast, the PSI of the function subscale was suboptimal (1.88), with a reliability of 0.78, suggesting this subscale may not be sensitive and precise enough to distinguish the quality of life between high and low performers. Item separation index (ISI) means different from the PSI, which is used to confirm the item difficulty hierarchy (= construct validity). Both subscales of the Rasch-revised AS-20 showed high ISI and reliability, suggesting that the sample size was big enough to precisely locate the items difficulty hierarchy on the different levels of quality of life.

The function subscale in its current form was not well matched to the Rasch model not only because of the unsatisfactory PSI and reliability, but also due to the need for revision of response options. Disordered response categories can occur when the neighboring categories may be indistinguishable to respondents and could be merged [21]. This was supported by Gothwal et al.’s findings that the response category did not function as expected for all items together in the AS-20. The reason they assumed was that the items of the AS-20 had too many response options for participants to distinguish between finer increments in response options. Therefore they reduced the response categories from the original 5-level to a new 3-level: ‘never’, ‘rarely/sometimes’ and ‘often/always’. They finally adopted the new 3-level rating scale since ideal PSI and targeting was achieved in the revised subscales. By comparison, in our study although the response categories in function subscale were properly oriented and distributed after the combination of categories, the PSR and targeting did not improve. Since the higher PSR mainly depended on wider sample ability variance, longer test and more response categories, the decreased PSR of the function subscale seemed reasonable after the combination and the number of the response categories was reduced. However, given that the unsatisfactory PSR has already presented before the re-category, the insufficient item number may underlie the unsatisfactory PSR in the function subscale in Chinese population.

The targeting provides an indication of how well targeted the items are for people in the sample by comparing of the mean score obtained for persons with that of the value of zero set for the items. The Rasch-revised psychosocial and function subscales demonstrated good targeting (i.e., the items are not too easy or too hard) of the items to the patients’ quality of life in our study cohort, because the mean location for strabismus patients were found around the value of zero (the mean value set for the items, 0.45 logits for the psychosocial subscale and 0.67 logits for the function one); in other words, the difference between the mean scores of the patients and the items were less than 1.0 logits. A positive mean value for the patients would indicate that the sample as a whole was located at a higher level of quality of life than the average of the scale.

## Conclusions

In conclusion, despite the need for re-categorization and unsatisfactory PSR in function subscale, the Chinese version of the AS-20 and its two subscales are generally reliable and valid tool among adult strabismus patients in China. Considering the lack of disease-specific QoL measurement for adult strabismus patients, undoubtedly this scale bridges the gap and provides a useful tool to evaluate the HRQoL in this patient population. It is noted that culture plays an important to interpret HRQoL; therefore culturally-appropriate validation becomes necessary when the AS-20 applying in a new ethnic population with different culture. Further studies are needed to test out assumption that the performance of the Chinese version of the AS-20 would improve if several items are added and new subdomains are established.

## Supporting Information

S1 Dataset A summary of DIF results. This file includes all tables and figures for the DIF results of all the different selected variables.
(DOCX)

S2 Dataset The original data of the 247 participants included in the study. This file includes the raw score of the AS-20 and participants’ demographic and clinical data.
(XLS)
