# Supplementary material for: Rasch Analysis of the Adult Strabismus Quality of Life Questionnaire (AS-20) among Chinese Adult Patients with Strabismus
Source: PLoS One. 2015 Nov 6;10(11):e0142188. doi: 10.1371/journal.pone.0142188 (PMC4636299; doi:10.1371/journal.pone.0142188)
Supplement: S1 Dataset — This file includes all tables and figures for the DIF results of all the different selected variables. (DOCX) [file pone.0142188.s001.docx]

Table 1. DIF of Gender for the AS-20 Psychosocial and Function Subscales

| **Item No.** | **Female** | |  | **Male** | | **DIF contrast** |
| --- | --- | --- | --- | --- | --- | --- |
|  | **DIF measure** | **SE** |  | **DIF measure** | **SE** |  |
| **Psychosocial**  **subscale** | |  |  |  |  |  |
| 1 | 0.92 | 0.11 |  | 0.83 | 0.11 | 0.09 |
| 2 | 0.02 | 0.11 |  | -0.08 | 0.11 | 0.10 |
| 3 | 0.90 | 0.11 |  | 0.83 | 0.11 | 0.07 |
| 4 | 0.44 | 0.11 |  | 0.25 | 0.11 | 0.18 |
| 5 | -0.49 | 0.11 |  | -0.45 | 0.11 | -0.05 |
| 6 | -0.18 | 0.11 |  | -0.25 | 0.11 | 0.08 |
| 7 | -1.05 | 0.12 |  | -0.91 | 0.12 | -0.14 |
| 8 | -0.40 | 0.11 |  | -0.35 | 0.11 | -0.05 |
| 9 | -0.39 | 0.11 |  | -0.23 | 0.11 | -0.16 |
| 10 | -0.45 | 0.11 |  | -0.24 | 0.11 | -0.20 |
| 17 | 0.65 | 0.11 |  | 0.60 | 0.11 | 0.05 |
| **Function**  **subscale** | |  |  |  |  |  |
| 11 | -0.55 | 0.11 |  | -0.50 | 0.10 | -0.05 |
| 12 | -0.73 | 0.11 |  | -0.91 | 0.11 | 0.18 |
| 13 | -0.43 | 0.10 |  | -0.33 | 0.10 | -0.10 |
| 14 | -0.50 | 0.10 |  | -0.44 | 0.10 | -0.06 |
| 15 | 0.72 | 0.09 |  | 0.53 | 0.09 | 0.18 |
| 16 | -0.68 | 0.11 |  | -0.40 | 0.10 | -0.28 |
| 18 | 1.57 | 0.10 |  | 1.50 | 0.11 | 0.07 |
| 19 | 0.53 | 0.09 |  | 0.53 | 0.09 | 0.00 |
| 20 | 0.04 | 0.10 |  | 0.04 | 0.10 | 0.00 |

Table 2. DIF of Age for the AS-20 Psychosocial and Function subscales

| **Item No.** | **≥Median age** | |  | **<Median age** | | **DIF contrast** |
| --- | --- | --- | --- | --- | --- | --- |
|  | **DIF measure** | **SE** |  | **DIF measure** | **SE** |  |
| **Psychosocial**  **subscale** | |  |  |  |  |  |
| 1 | 0.75 | 0.12 |  | 0.95 | 0.10 | -0.21 |
| 2 | 0.11 | 0.12 |  | -0.12 | 0.10 | 0.22 |
| 3 | 0.75 | 0.12 |  | 0.94 | 0.10 | -0.19 |
| 4 | 0.25 | 0.12 |  | 0.40 | 0.10 | -0.15 |
| 5 | -0.26 | 0.12 |  | -0.60 | 0.10 | 0.34 |
| 6 | -0.26 | 0.12 |  | -0.18 | 0.10 | -0.08 |
| 7 | -0.88 | 0.13 |  | -1.04 | 0.11 | 0.16 |
| 8 | -0.33 | 0.12 |  | -0.40 | 0.10 | 0.08 |
| 9 | -0.28 | 0.12 |  | -0.31 | 0.10 | 0.03 |
| 10 | -0.40 | 0.12 |  | -0.31 | 0.10 | -0.09 |
| 17 | 0.57 | 0.12 |  | 0.66 | 0.10 | -0.09 |
| **Function**  **subscale** | |  |  |  |  |  |
| 11 | -0.64 | 0.12 |  | -0.45 | 0.09 | -0.19 |
| 12 | -0.66 | 0.12 |  | -0.94 | 0.11 | 0.29 |
| 13 | -0.38 | 0.11 |  | -0.38 | 0.09 | 0.00 |
| 14 | -0.50 | 0.12 |  | -0.44 | 0.09 | -0.06 |
| 15 | 0.63 | 0.11 |  | 0.63 | 0.08 | 0.00 |
| 16 | -0.33 | 0.11 |  | -0.67 | 0.10 | 0.34 |
| 18 | 1.47 | 0.12 |  | 1.58 | 0.09 | -0.12 |
| 19 | 0.43 | 0.11 |  | 0.59 | 0.08 | -0.16 |
| 20 | 0.04 | 0.11 |  | 0.04 | 0.09 | 0.00 |

Table 3. DIF of Education level for the AS-20 Psychosocial and Function subscales

| **Item No.** | **High level of education** | |  | **Low level of education** | | **DIF contrast** |
| --- | --- | --- | --- | --- | --- | --- |
|  | **DIF measure** | **SE** |  | **DIF measure** | **SE** |  |
| **Psychosocial**  **subscale** | |  |  |  |  |  |
| 1 | 0.93 | 0.09 |  | 0.78 | 0.13 | 0.15 |
| 2 | -0.08 | 0.10 |  | 0.05 | 0.13 | -0.13 |
| 3 | 0.95 | 0.09 |  | 0.70 | 0.13 | 0.26 |
| 4 | 0.48 | 0.09 |  | 0.10 | 0.13 | 0.39 |
| 5 | -0.47 | 0.10 |  | -0.47 | 0.13 | 0.00 |
| 6 | -0.15 | 0.10 |  | -0.33 | 0.13 | 0.18 |
| 7 | -1.14 | 0.11 |  | -0.72 | 0.13 | -0.42 |
| 8 | -0.47 | 0.10 |  | -0.22 | 0.13 | -0.25 |
| 9 | -0.31 | 0.10 |  | -0.31 | 0.13 | 0.00 |
| 10 | -0.48 | 0.10 |  | -0.13 | 0.13 | -0.35 |
| 17 | 0.66 | 0.09 |  | 0.57 | 0.13 | 0.09 |
| **Function**  **subscale** | |  |  |  |  |  |
| 11 | -0.53 | 0.09 |  | -0.53 | 0.12 | 0.00 |
| 12 | -0.88 | 0.10 |  | -0.73 | 0.13 | -0.15 |
| 13 | -0.35 | 0.09 |  | -0.44 | 0.12 | 0.09 |
| 14 | -0.38 | 0.09 |  | -0.62 | 0.12 | 0.25 |
| 15 | 0.63 | 0.08 |  | 0.63 | 0.11 | 0.00 |
| 16 | -0.63 | 0.10 |  | -0.38 | 0.12 | -0.25 |
| 18 | 1.54 | 0.09 |  | 1.50 | 0.12 | 0.04 |
| 19 | 0.53 | 0.08 |  | 0.53 | 0.11 | 0.00 |
| 20 | 0.04 | 0.08 |  | 0.04 | 0.11 | 0.00 |

Table 4. DIF of Strabismus type for the AS-20 Psychosocial and Function subscales

| **Item No.** | **Exotropia** | |  | **Esotropia** | | **DIF contrast** |
| --- | --- | --- | --- | --- | --- | --- |
|  | **DIF measure** | **SE** |  | **DIF measure** | **SE** |  |
| **Psychosocial**  **subscale** | |  |  |  |  |  |
| 1 | 0.83 | 0.09 |  | 0.98 | 0.14 | -0.15 |
| 2 | 0.02 | 0.09 |  | -0.14 | 0.14 | 0.16 |
| 3 | 0.86 | 0.09 |  | 0.82 | 0.14 | 0.04 |
| 4 | 0.26 | 0.09 |  | 0.56 | 0.14 | -0.30 |
| 5 | -0.44 | 0.09 |  | -0.55 | 0.14 | 0.11 |
| 6 | -0.29 | 0.09 |  | -0.04 | 0.14 | -0.25 |
| 7 | -0.98 | 0.10 |  | -1.01 | 0.15 | 0.03 |
| 8 | -0.39 | 0.09 |  | -0.33 | 0.14 | -0.06 |
| 9 | -0.24 | 0.09 |  | -0.47 | 0.14 | 0.23 |
| 10 | -0.34 | 0.09 |  | -0.38 | 0.14 | 0.03 |
| 17 | 0.67 | 0.09 |  | 0.54 | 0.14 | 0.13 |
| **Function**  **subscale** | |  |  |  |  |  |
| 11 | -0.60 | 0.09 |  | -0.35 | 0.13 | -0.25 |
| 12 | -0.82 | 0.09 |  | -0.80 | 0.14 | -0.02 |
| 13 | -0.34 | 0.08 |  | -0.49 | 0.13 | 0.16 |
| 14 | -0.50 | 0.09 |  | -0.39 | 0.13 | -0.12 |
| 15 | 0.68 | 0.08 |  | 0.50 | 0.12 | 0.17 |
| 16 | -0.51 | 0.09 |  | -0.58 | 0.14 | 0.07 |
| 18 | 1.54 | 0.09 |  | 1.57 | 0.13 | -0.03 |
| 19 | 0.48 | 0.08 |  | 0.65 | 0.12 | -0.17 |
| 20 | 0.10 | 0.08 |  | -0.09 | 0.13 | 0.18 |


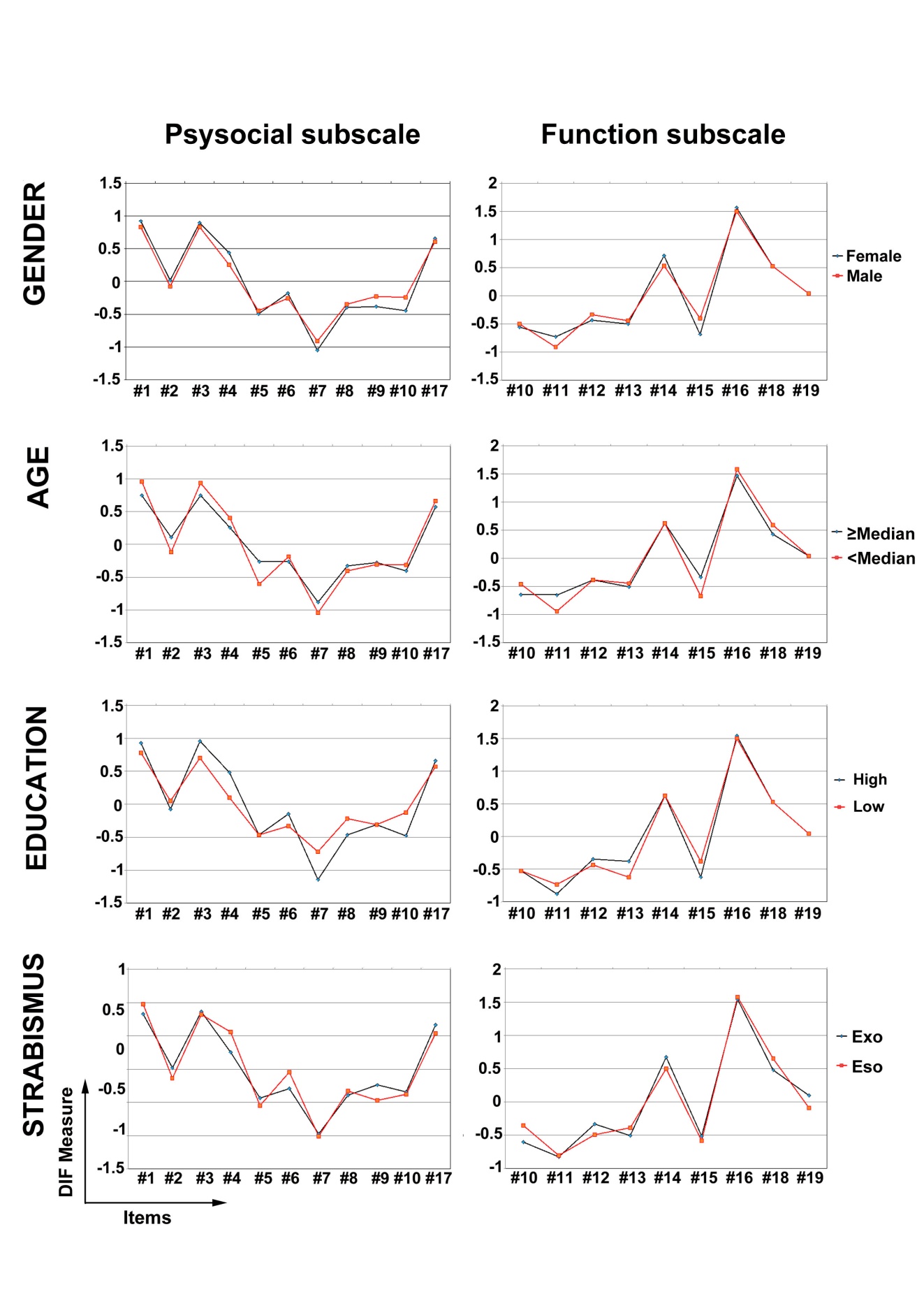


Figure 1. DIF plot showing measures regarding to different variables: gender, age, education and strabismus type.

Table 5. DIF of Living areas for the AS-20 Psychosocial and Function subscales

| **Item No.** | **Rural** | |  | **Urban** | | **DIF contrast** |
| --- | --- | --- | --- | --- | --- | --- |
|  | **DIF measure** | **SE** |  | **DIF measure** | **SE** |  |
| **Psychosocial**  **subscale** | |  |  |  |  |  |
| 1 | 0.94 | 0.12 |  | 0.82 | 0.10 | 0.12 |
| 2 | -0.03 | 0.11 |  | -0.03 | 0.10 | 0.00 |
| 3 | 0.74 | 0.11 |  | 0.96 | 0.10 | -0.21 |
| 4 | 0.21 | 0.11 |  | 0.46 | 0.10 | -0.25 |
| 5 | -0.47 | 0.12 |  | -0.47 | 0.10 | 0.00 |
| 6 | -0.23 | 0.11 |  | -0.21 | 0.10 | -0.02 |
| 7 | -1.10 | 0.13 |  | -0.88 | 0.11 | -0.22 |
| 8 | -0.31 | 0.12 |  | -0.42 | 0.10 | 0.11 |
| 9 | -0.31 | 0.12 |  | -0.31 | 0.10 | 0.00 |
| 10 | -0.30 | 0.12 |  | -0.38 | 0.10 | 0.08 |
| 17 | 0.81 | 0.11 |  | 0.49 | 0.10 | 0.32 |
| **Function**  **subscale** | |  |  |  |  |  |
| 11 | -0.53 | 0.11 |  | -0.53 | 0.10 | 0.00 |
| 12 | -0.86 | 0.12 |  | -0.79 | 0.10 | -0.07 |
| 13 | -0.41 | 0.11 |  | -0.35 | 0.09 | -0.06 |
| 14 | -0.57 | 0.11 |  | -0.39 | 0.09 | -0.18 |
| 15 | 0.63 | 0.10 |  | 0.63 | 0.09 | 0.00 |
| 16 | -0.44 | 0.11 |  | -0.61 | 0.10 | 0.17 |
| 18 | 1.68 | 0.12 |  | 1.44 | 0.09 | 0.24 |
| 19 | 0.47 | 0.10 |  | 0.58 | 0.09 | -0.11 |
| 20 | 0.08 | 0.10 |  | 0.02 | 0.09 | 0.06 |

Table 6. DIF of Family support for the AS-20 Psychosocial and Function subscales

| **Item No.** | **Never & Sometimes** | |  | **Always** | | **DIF contrast** |
| --- | --- | --- | --- | --- | --- | --- |
|  | **DIF measure** | **SE** |  | **DIF measure** | **SE** |  |
| **Psychosocial**  **subscale** | |  |  |  |  |  |
| 1 | 0.87 | 0.10 |  | 0.87 | 0.12 | 0.00 |
| 2 | -0.07 | 0.10 |  | 0.03 | 0.12 | -0.10 |
| 3 | 0.80 | 0.10 |  | 0.96 | 0.12 | -0.17 |
| 4 | 0.32 | 0.10 |  | 0.38 | 0.12 | -0.06 |
| 5 | -0.41 | 0.10 |  | -0.55 | 0.12 | 0.14 |
| 6 | -0.21 | 0.10 |  | -0.24 | 0.12 | 0.03 |
| 7 | -0.93 | 0.11 |  | -1.06 | 0.13 | 0.13 |
| 8 | -0.31 | 0.10 |  | -0.46 | 0.12 | 0.15 |
| 9 | -0.31 | 0.10 |  | -0.33 | 0.12 | 0.02 |
| 10 | -0.37 | 0.10 |  | -0.30 | 0.12 | -0.07 |
| 17 | 0.59 | 0.10 |  | 0.69 | 0.12 | -0.10 |
| **Function**  **subscale** | |  |  |  |  |  |
| 11 | -0.67 | 0.10 |  | -0.31 | 0.11 | -0.36 |
| 12 | -0.74 | 0.10 |  | -0.96 | 0.13 | 0.23 |
| 13 | -0.33 | 0.09 |  | -0.46 | 0.12 | 0.12 |
| 14 | -0.40 | 0.09 |  | -0.58 | 0.12 | 0.18 |
| 15 | 0.56 | 0.08 |  | 0.74 | 0.11 | -0.18 |
| 16 | -0.43 | 0.09 |  | -0.70 | 0.12 | 0.27 |
| 18 | 1.50 | 0.09 |  | 1.61 | 0.12 | -0.11 |
| 19 | 0.46 | 0.08 |  | 0.64 | 0.11 | -0.18 |
| 20 | 0.09 | 0.09 |  | -0.03 | 0.11 | 0.12 |

Table 7. DIF of Diplopia for the AS-20 Psychosocial and Function subscales

| **Item No.** | **Diplopia** | |  | **Non-diplopia** | | **DIF contrast** |
| --- | --- | --- | --- | --- | --- | --- |
|  | **DIF measure** | **SE** |  | **DIF measure** | **SE** |  |
| **Psychosocial**  **subscale** | |  |  |  |  |  |
| 1 | 0.87 | 0.13 |  | 0.87 | 0.10 | 0.00 |
| 2 | -0.15 | 0.13 |  | 0.04 | 0.09 | -0.19 |
| 3 | 0.90 | 0.13 |  | 0.86 | 0.10 | 0.03 |
| 4 | 0.35 | 0.12 |  | 0.35 | 0.09 | 0.00 |
| 5 | -0.58 | 0.13 |  | -0.41 | 0.10 | -0.17 |
| 6 | -0.04 | 0.13 |  | -0.32 | 0.10 | 0.27 |
| 7 | -0.91 | 0.14 |  | -1.02 | 0.10 | 0.10 |
| 8 | -0.35 | 0.13 |  | -0.37 | 0.10 | 0.03 |
| 9 | -0.43 | 0.13 |  | -0.24 | 0.10 | -0.19 |
| 10 | -0.37 | 0.13 |  | -0.34 | 0.10 | -0.02 |
| 17 | 0.72 | 0.13 |  | 0.58 | 0.09 | 0.15 |
| **Function**  **subscale** | |  |  |  |  |  |
| 11 | -0.41 | 0.12 |  | -0.60 | 0.09 | 0.18 |
| 12 | -0.80 | 0.13 |  | -0.82 | 0.10 | 0.02 |
| 13 | -0.33 | 0.12 |  | -0.41 | 0.09 | 0.08 |
| 14 | -0.47 | 0.12 |  | -0.47 | 0.09 | 0.00 |
| 15 | 0.58 | 0.11 |  | 0.65 | 0.08 | -0.06 |
| 16 | -0.57 | 0.12 |  | -0.51 | 0.09 | -0.06 |
| 18 | 1.54 | 0.12 |  | 1.54 | 0.09 | 0.00 |
| 19 | 0.49 | 0.11 |  | 0.55 | 0.08 | -0.07 |
| 20 | 0.00 | 0.11 |  | 0.07 | 0.08 | -0.07 |

Table 8. DIF of Insurance support for the AS-20 Psychosocial and Function subscales

| **Item No.** | **Presence of insurance support** | |  | **Absence of insurance support** | | **DIF contrast** |
| --- | --- | --- | --- | --- | --- | --- |
|  | **DIF measure** | **SE** |  | **DIF measure** | **SE** |  |
| **Psychosocial**  **subscale** | |  |  |  |  |  |
| 1 | 0.82 | 0.13 |  | 0.90 | 0.09 | -0.08 |
| 2 | 0.00 | 0.13 |  | -0.03 | 0.09 | 0.03 |
| 3 | 0.86 | 0.13 |  | 0.86 | 0.09 | 0.00 |
| 4 | 0.28 | 0.13 |  | 0.38 | 0.09 | -0.10 |
| 5 | -0.44 | 0.14 |  | -0.47 | 0.09 | 0.03 |
| 6 | -0.13 | 0.13 |  | -0.26 | 0.09 | 0.13 |
| 7 | -1.17 | 0.15 |  | -0.90 | 0.10 | -0.27 |
| 8 | -0.37 | 0.14 |  | -0.37 | 0.09 | 0.00 |
| 9 | -0.23 | 0.13 |  | -0.34 | 0.09 | 0.11 |
| 10 | -0.20 | 0.13 |  | -0.42 | 0.09 | 0.22 |
| 17 | 0.54 | 0.13 |  | 0.67 | 0.09 | -0.13 |
| **Function**  **subscale** | |  |  |  |  |  |
| 11 | -0.59 | 0.14 |  | -0.50 | 0.09 | -0.08 |
| 12 | -0.82 | 0.14 |  | -0.82 | 0. 09 | 0.00 |
| 13 | -0.38 | 0.13 |  | -0.38 | 0. 09 | 0.00 |
| 14 | -0.60 | 0.14 |  | -0.41 | 0. 09 | -0.19 |
| 15 | 0.55 | 0.12 |  | 0.66 | 0.08 | -0.11 |
| 16 | -0.74 | 0.14 |  | -0.45 | 0. 09 | -0.29 |
| 18 | 1.87 | 0.14 |  | 1.40 | 0. 09 | 0.48 |
| 19 | 0.64 | 0.12 |  | 0.48 | 0.08 | 0.16 |
| 20 | 0.04 | 0.12 |  | 0.04 | 0.08 | 0.00 |


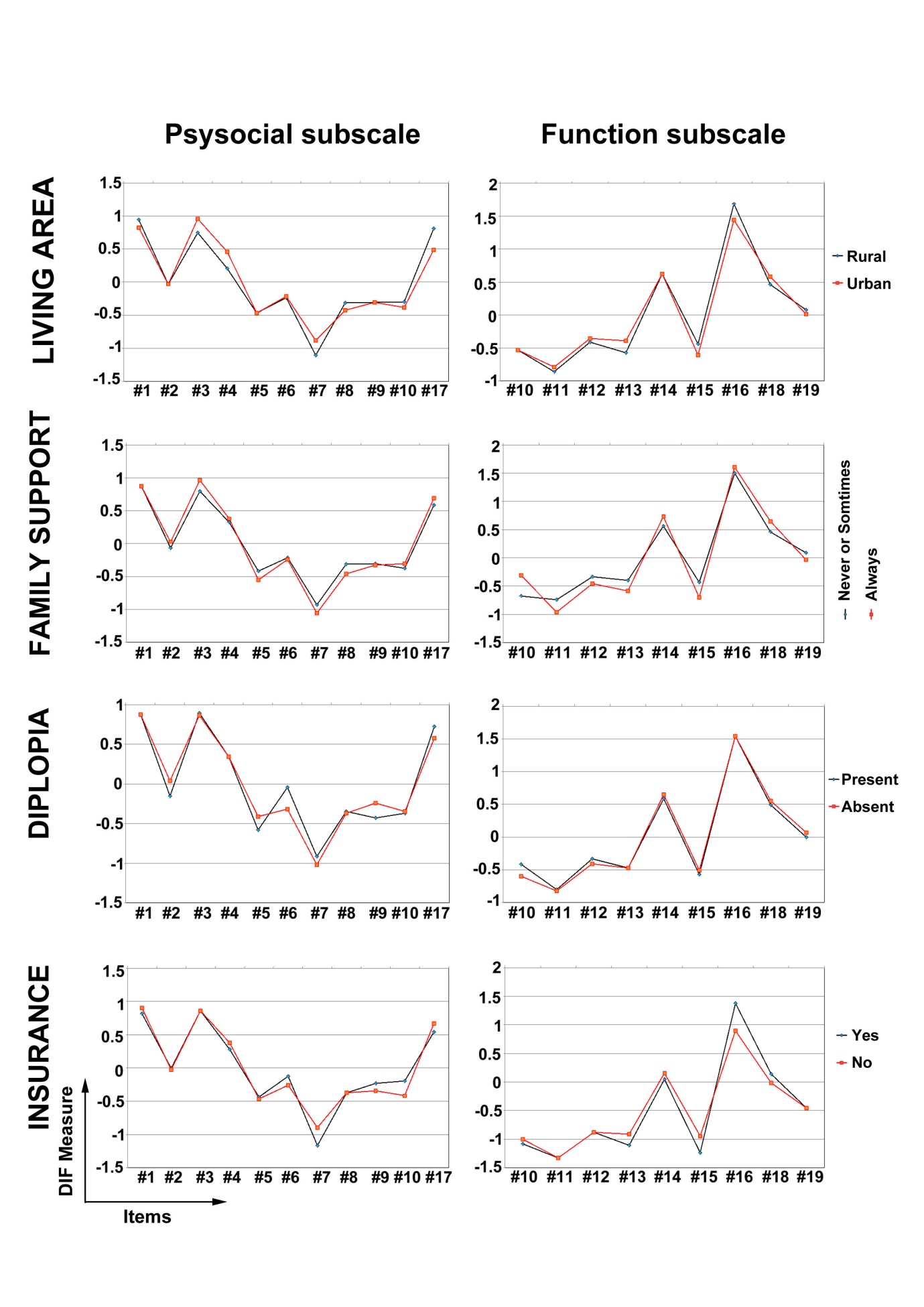


Figure 2. DIF plot showing measures regarding to different variables: living area, family support, diplopia and insurance support
